# Teaching Ultrasound-Guided Peripheral Cannulation to Doctors in the UK: A Quality Improvement, Feasibility, and Impact Evaluation

**DOI:** 10.7759/cureus.99248

**Published:** 2025-12-15

**Authors:** Isobel S Burridge, Benjamin Samra, Stephen Cole

**Affiliations:** 1 Trauma and Orthopedics, Huddersfield Royal Infirmary, Huddersfield, GBR; 2 General Surgery, University Hospitals Birmingham NHS Foundation Trust, Birmingham, GBR; 3 Intensive Care, Buckinghamshire Healthcare NHS Trust, High Wycombe, GBR

**Keywords:** medical education, peripheral intravenous access, simulation-based training, skill acquisition, ultrasound-guided cannulation

## Abstract

Introduction

Timely peripheral IV access is essential for delivering lifesaving treatments such as IV antibiotics in septic patients. However, many doctors lack confidence and technical proficiency in ultrasound (US)-guided cannulation, which may contribute to treatment delays and unnecessary escalation to senior or anesthetic colleagues.

Methods

As part of a quality improvement initiative, a single-center pre-post educational evaluation without a control group was conducted to assess the impact of a brief US-guided peripheral IV cannulation (US-PIVC) teaching intervention on clinicians’ self-reported confidence and subsequent translation into clinical practice. A reproducible one-hour mixed-modality session combining concise didactic instruction with supervised hands-on practice using Blue Phantom 2-Vessel Vascular Block phantoms was delivered. US machines equipped with high-frequency linear probes were used. The primary outcome was change in self-reported confidence, measured using a 10-point Likert scale across six domains. Secondary outcomes included self-reported clinical application, escalation to senior or anesthetic colleagues, and perceived need for further phantom practice at three months. Baseline surveys gathered information regarding escalation for difficult cannulations and perceived treatment delays. Confidence was measured immediately before and after training, and a three-month follow-up survey assessed subsequent clinical use.

Results

Twelve sessions trained 84 doctors. Baseline responses (n = 67) indicated that 83.6% had escalated difficult cannulations to senior or anesthetic colleagues in the preceding six months, and 91.0% perceived delays in patient care due to IV access difficulties. Pre-session confidence across all domains was low (mean scores, 1.21/10-4.61/10). Post-session confidence increased markedly to mean scores of 8.96/10-10.00/10. Overall confidence in using the US to aid cannulation increased from 1.35 ± 0.89 to 9.23 ± 1.22. At the three-month follow-up (n = 35; 41.7% response rate), 74.3% reported successful clinical use of US-PIVC, 88.6% felt no additional phantom practice was needed, and 5.7% had required escalation; however, baseline and follow-up escalation data were derived from different respondent groups and therefore do not represent a matched comparison.

Conclusions

A single one-hour mixed-modality US-PIVC teaching session, delivered using existing hospital resources, was associated with substantial improvements in self-reported confidence and self-reported clinical application among respondents. As the primary outcome was subjective confidence and no objective performance or patient-level outcomes were collected, the clinical impact should be interpreted cautiously. Brief, structured US-PIVC teaching sessions may enhance perceived procedural independence if embedded early in training, although further evaluation incorporating objective metrics is required to confirm clinical benefit.

## Introduction

Peripheral IV cannulation (PIVC) is among the most frequently performed invasive procedures in healthcare, yet difficulty in obtaining IV access remains a common clinical challenge. Failed attempts can cause significant treatment delays, repeated needle trauma, and unnecessary escalation to senior or anesthetic colleagues, increasing workload and diverting resources from other acute care duties [1,2]. Risk factors for difficult cannulation include obesity, chronic illness, IV drug use, and prior chemotherapy, among others [3]. Timely IV access is particularly critical in the management of sepsis, where the National Institute for Health and Care Excellence (NICE) guideline NG51 (2024) recommends administration of IV antibiotics within one hour of recognition to optimize outcomes [4].

Ultrasound-guided PIVC (US-PIVC) has been shown to improve procedural success, reduce insertion attempts, and increase patient comfort when compared with traditional landmark techniques [2-5]. Despite these advantages, US-guided cannulation training is inconsistently incorporated into medical education programs, resulting in variable levels of clinician proficiency and continued reliance on ad hoc teaching [6]. Educational barriers, rather than equipment availability, have been recognized as the principal obstacle to widespread adoption [6,7].

Previous work, including that by Hoskins et al. (2023), has demonstrated that structured mixed-modality teaching combining concise theoretical instruction with supervised simulation can improve procedural knowledge, technical skill, and confidence, independent of prior experience [8]. Similarly, studies by Lian et al. (2017) and Gleeson et al. (2024) support the effectiveness of brief and focused teaching interventions that incorporate simulation and feedback [5,7].

To address an identified skills gap at the John Radcliffe Hospital in Oxford, a quality improvement (QI) project was initiated to develop and implement a structured one-hour US-guided cannulation teaching session. The intervention integrated concise didactic instruction with supervised hands-on practice using tissue-mimicking vascular access phantoms. The objective of this educational evaluation was to assess the feasibility of delivering such training using existing hospital resources and to determine its impact on clinicians’ self-reported confidence and subsequent application in clinical practice.

## Materials and methods

Study design and setting

This study used a single-center pre-post educational evaluation design within a quality-improvement framework, without a control group. The project was conducted at the John Radcliffe Hospital (Oxford, UK) over a seven-month period in 2023. The project was registered as a QI initiative, which confirmed that formal research ethics committee approval was not required. Participation was voluntary, and completion of questionnaires was considered implied consent.

Resources and setup

Teaching sessions required US machines and tissue-mimicking vascular access phantoms. The phantoms used were the Blue Phantom 2-Vessel Vascular Block models, manufactured by Elevate Healthcare (Sarasota, Florida, USA), which replicate soft tissue and vascular structures for safe practice, and were provided by the University of Oxford Clinical Skills Laboratory [9]. Additional US machines equipped with high-frequency linear probes were borrowed from the Emergency Department education center and other clinical areas. Sessions were scheduled during lunchtime to minimize disruption to clinical duties and maintained an optimal learner-to-machine ratio of approximately 2:1. Up to two additional participants were occasionally accommodated to account for clinical absences.

Three trainers facilitated the program. To minimize variability, all trainers used the same Google Slides (Google LLC, Mountain View, CA, USA) and an agreed-upon script for the didactic component, and each trainer observed another teach over the course of the program. Although individual teaching styles naturally differed, the use of standardized materials and peer observation ensured that the content delivered was consistent and that variation in information provided to participants was minimal.

Participant recruitment 

Participants were recruited through foundation program mailing lists, departmental rota coordinators within medical, surgical, and emergency medicine teams, and local WhatsApp groups. Interested doctors were invited to sign up via an accessible Google document and were asked to provide their name and email address. The form could be freely shared between colleagues to allow registration by those not included on existing mailing lists. Sessions consistently reached full capacity within twenty-four hours of release.

Teaching structure 

Each session accommodated eight to 10 participants and followed a mixed-modality design. The didactic component, lasting approximately 20 to 30 minutes, was delivered through a PowerPoint presentation covering the principles of US physics and image generation, probe selection and machine orientation, adjustment of gain and depth, differentiation of veins from arteries, identification of suitable veins, in-plane and out-of-plane approaches, tourniquet application, and dynamic needle tip positioning using the “target sign” technique.

The practical component, lasting 30 to 40 minutes, consisted of hands-on practice in pairs using Blue Phantom 2-Vessel Vascular Block phantoms. Participants scanned each other to identify target vessels before practicing guided needle placement on the phantoms under facilitator supervision. Attendees were encouraged to remain until confident, allowing flexible learning within the one-hour time frame.

Evaluation and data collection 

Data were collected at three time points using online surveys (prior to the session and at the three-month follow-up) and paper questionnaires (pre- and post-session). The primary outcome was the change in self-reported confidence across six domains of US-PIVC. Secondary outcomes were self-reported clinical use of US-PIVC, escalation to senior or anesthetic colleagues for difficult cannulations, and perceived need for further phantom practice at three months.

The pre-session survey, distributed via Google Forms (Google LLC) 48 hours before the workshop to the email addresses provided during registration, recorded the participants’ level of training, whether they had recently escalated difficult cannulations to senior or anesthetic colleagues, and whether they perceived treatment delays due to IV access difficulties.

Pre- and post-session confidence was assessed using a 10-point Likert scale, where 1 indicated low confidence and 10 indicated high confidence. Participants rated their confidence across six domains: selecting the correct probe for US-guided cannulation, adjusting and understanding gain, adjusting and understanding depth, identifying relevant anatomy, including veins and arteries, understanding in-plane versus out-of-plane techniques, and overall comfort using US to aid cannulation.

A follow-up survey was distributed three months after the session via Google Forms to assess skill retention and application in clinical practice. Participants were asked whether they had successfully used the technique in practice, whether they had required escalation to senior or anesthetic colleagues, and whether they desired further phantom-based practice.

We did not systematically collect data on prior US experience, trainer identity, or individual clinical access to US machines. These factors are therefore potential confounders and are addressed in the Limitations section.

## Results

Twelve sessions were delivered over a seven-month period in 2023. The size of each group was limited by the number of US machines available, and the frequency of sessions was constrained by faculty availability. A total of 84 participants attended across all sessions.

Baseline data (pre-session Google Form) 

Prior to the teaching sessions, 67 participants completed the baseline questionnaire. Respondents comprised 19 Foundation Year 1 doctors, 33 Foundation Year 2 doctors, 12 core trainees or equivalent, three specialty registrars or equivalent, and one consultant. 

The majority (83.6%; n=56) reported escalating difficult cannulations to senior or anesthetic colleagues within the preceding six months. Furthermore, 61 participants (91.0%) indicated that difficulties with cannula insertion had resulted in delays to patient care.

Pre-session confidence questionnaire 

**Figure 1:**
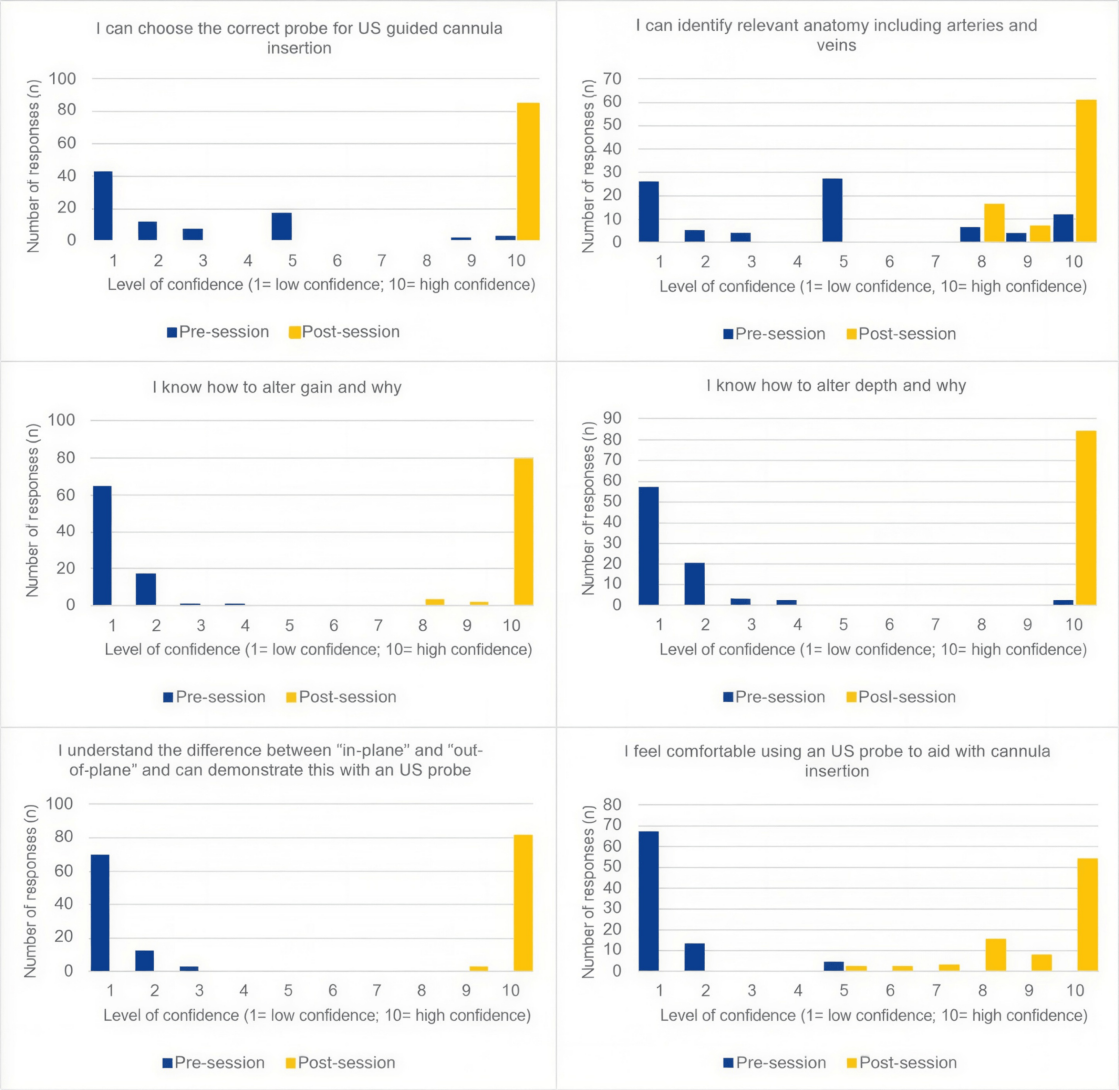
Statements shown to participants alongside their self-reported confidence feedback scores, rated on a scale from 1 (lowest confidence) to 10 (highest confidence)

The pre-session confidence questionnaire demonstrated low baseline confidence across all domains (Figure 1). The mean pre-session score for feeling comfortable using a US probe to aid cannula insertion was 1.35 ± 0.89. Similarly, participants reported limited confidence in selecting the appropriate probe (2.63 ± 2.34), adjusting gain (1.26 ± 0.54), altering depth (1.57 ± 1.34), and understanding in-plane versus out-of-plane techniques (1.21 ± 0.49). The highest baseline confidence was observed in identifying relevant anatomy (veins and arteries), with a mean score of 4.61 ± 3.24.

Post-session confidence questionnaire 

The post-session questionnaire demonstrated marked improvement across all domains. Confidence in selecting the appropriate probe rose to 10.00 ± 0.00, knowledge of adjusting gain improved to 9.85 ± 0.62, confidence in adjusting depth reached 10.00 ± 0.00, understanding of in-plane versus out-of-plane techniques increased to 8.96 ± 0.18, and identifying relevant anatomy increased to 9.54 ± 0.79. The mean post-session score for comfort using a US probe increased to 9.23 ± 1.22.

Three-month follow-up (post-session Google Form)

At the three-month follow-up, 35 participants completed the questionnaire. Of these, 26 (74.3%) reported successfully applying their newly acquired US-guided cannulation skills in clinical practice. The remaining nine participants (25.7%) had not yet used the technique, most commonly due to a lack of opportunity (n = 5) or limited access to a US machine (n = 4).

Only two respondents (5.7%) had required escalation to senior or anesthetic colleagues since training. The majority (88.6%; n = 31) felt no additional phantom practice was necessary, suggesting durable confidence and skill retention following the teaching intervention.

## Discussion

This QI evaluation demonstrates that a brief, structured US-guided cannulation teaching session can substantially improve self-reported confidence among doctors and is feasible to deliver using existing hospital resources. The intervention proved both popular and reproducible, reflecting its relevance to clinical practice and its potential for wider implementation.

The pre-session questionnaire revealed a clear deficit in US-PIVC confidence and experience among resident doctors. Most participants had previously escalated difficult cannulations to senior or anesthetic colleagues, and the majority reported treatment delays related to IV access. These findings highlight an important educational need, particularly in the context of sepsis management, where NICE NG51 guidelines emphasize antibiotic administration within one hour of recognition [4].

Delivering sessions during lunchtime maximized accessibility and attendance without disrupting clinical activity. Consistent oversubscription demonstrated both the practicality of this scheduling approach and the strong perceived value of the training among doctors.

The teaching intervention proved highly effective at improving self-reported confidence. A single, one-hour session combining concise theory with supervised practical training resulted in significant improvement across all confidence domains, with mean post-session scores approaching 10/10. This outcome supports existing literature demonstrating that brief, structured, mixed-modality sessions can rapidly enhance procedural competence [8].

Translation of learning into clinical practice was evident at three-month follow-up, with 74.3% of respondents reporting successful application of their new skills in patient care. Only two participants (5.7%) required escalation to senior colleagues, both citing time constraints rather than lack of confidence. This represents a substantial reduction from the 83.6% escalation rate reported before the session, suggesting meaningful behavioral change and increased independence. Sustained confidence and low demand for additional practice sessions indicate long-term impact.

The model’s feasibility and scalability are key strengths. By utilizing existing hospital resources, including skills lab phantoms and departmental US machines, this program required minimal financial outlay. However, it is important to acknowledge that phantoms remain a relatively costly resource and must be sourced or borrowed by facilitators. In addition, reliance on departmental or representative support to access US machines may limit the frequency or sustainability of sessions in some settings. Addressing these logistical challenges through departmental investment or shared equipment models could further enhance the availability of such teaching programs. 

This intervention adds to the existing literature by demonstrating that a brief, low-cost, and scalable model can be delivered using resources already available within most hospitals. The consistently high engagement and strong demand observed across twelve fully subscribed sessions, without exhausting the pool of interested participants, further underscores the substantial training gap in US-PIVC skills within our institution. Our findings, therefore, highlight both the degree of unmet educational need and the potential of this focused program to improve clinicians’ confidence in using US-PIVC.

Several limitations should be acknowledged. The study relied on self-reported confidence and follow-up survey data, both of which are subjective and susceptible to social desirability and Hawthorne effects. The low three-month follow-up rate raises the possibility of responder bias because participants who responded may differ systematically from non-responders, potentially favoring those who were more motivated or more likely to use US-PIVC successfully. Baseline escalation rates and follow-up escalation reports were not matched to respondents and, therefore, cannot be interpreted as a direct comparison. Confounders such as prior US experience, trainer variability, and differential access to US machines were not formally measured and may have influenced the results. The single-center design and modest sample size further limit external validity.

Future work should aim to quantify the clinical impact of US-PIVC training by assessing measurable outcomes such as reduced anesthetic referrals, shorter treatment delays, and improved patient care metrics. Competence should be evaluated using objective performance measures, including first-pass success rate, procedure time, and formal skills assessment. Demonstrating cost-effectiveness and tangible clinical benefit would strengthen the case for institutional investment in training delivery and in the procurement and maintenance of essential equipment such as US machines and phantoms. Such evidence would further support the inclusion of US-PIVC teaching within formal foundation and specialty curricula, ensuring consistent exposure, improved skill retention, and the establishment of this technique as a core procedural competency among doctors.

## Conclusions

This study demonstrates that a concise, structured teaching session can effectively increase doctors’ self-reported confidence in US-guided peripheral cannulation. A single one-hour intervention combining targeted theory with hands-on supervision led to substantial confidence gains and was associated with self-reported use in clinical practice among responders. Because the study did not assess objective competence or patient outcomes, these findings should be interpreted as improvements in perceived capability rather than confirmed clinical performance. The program’s use of existing hospital resources highlights its feasibility and scalability. Embedding such training early in medical education may enhance clinicians’ perceived procedural independence, with future studies needed to determine its impact on competence and patient care outcomes.
